# Gout and Its Cure

**Published:** 1895-08

**Authors:** 


GOUT AND ITS CURE. By J. Compton Burnett, M. D. Philadelphia: Boericke & Tafel. 1895. Price, cloth, by mail, 90 cents, net. By mail, 95 cents.

This book is the latest addition to the library of clever monographs by that agreeable writer, Dr. Burnett.

The writer considers that there are two kinds of gout—surfeit gout and exhaustion gout.

Surfeit gout must be treated by abstemiousness, and the author has seen "attacks of this kind of goût brought on by a single bottle of beer or a pint of even the dryest champagne, and on one occasion from eating gooseberries."

Exhaustion gout, on the other hand, needs stimulants and generous diet. Thus the author saw a case of this kind of gout which had been brought on by the spare diet prescribed by a certain antifat doctor.

If there are cases which appear to be due to surfeit, and so need abstinence, and other cases due to exhaustion, and so need stimulation, there are also cases which seem to partake of both qualities, surfeit and exhaustion. These cases puzzle the physician, who must choose between stimulation and abstinence in his treatment.

In this dilemma he simply stimulates and tries to get a favorable result from variation in the character of the stimulant.

Thus he allows such patients "a small quantity of dry champagne for dinner once or twice a week, the same quantity of a sound claret once or twice a week, and whiskey and water, or whiskey and soda-water, on other days."

To a man up a tree gazing on the landscape through homœopathic glasses, this declaration comes as a genuine surprise from a homœopathic physician. Especially as over in one corner of the field he may see a little group of strict simillimum fiends curing their cases by just earnestly seeking the similar remedy and ignoring these pathological suggestions that lead up to rational treatment.

Dr. Burnett also resorts to the similar remedy, and he mentions over the remedies that have been beneficial. They are Natrum-muriaticum, 6th trituration; Aurum-muriaticum, the third decimal; Aurum-muriaticum-naturatum, third decimal; Jaborandi and its alkaloid Pilocarpine, and lastly, and most important, Urtica-urens. This drug is regarded by Dr. Burnett as by far the most important medicine that can be used in the treatment of gout.

Patients under the influence of Urtica-urens in small material doses passed large quantities of gravel in the urine, and in one case related, gravel appeared in the stool.

The indications for it in gout are the presence of gravel in the urine; fever, coming on at night; a previous history of malaria; *pain in the region of the spleen;* vertigo, with tendency to fall forward, and headache.

The key-note to these cases of gout seems to be the pain in the region of the spleen.

Much more is said than is here stated; and it is said in the most captivating manner, too. This makes it more impressive; but we have not the space to reproduce these statements. As this review hardly does justice to the book, all who are interested should get their ideas of it clear by reading it for themselves.

